# Additive Manufacturing for Guided Bone Regeneration: A Perspective for Alveolar Ridge Augmentation

**DOI:** 10.3390/ijms19113308

**Published:** 2018-10-24

**Authors:** Patrick Rider, Željka Perić Kačarević, Said Alkildani, Sujith Retnasingh, Reinhard Schnettler, Mike Barbeck

**Affiliations:** 1 Botiss Biomaterials GmbH, Hauptstr. 28, 15806 Zossen, Germany; 2 Department of Anatomy, Histology and Embryology, Faculty of Dental Medicine and Health, Josip Juraj Strossmayer University of Osijek, Osijek 31000, Croatia; Zeljka.Peric.Kacarevic@botiss.com; 3 Department of Biomedical Engineering, Faculty of Applied Medical Sciences, German-Jordanian University, Amman 11180, Jordan; saidkildani@gmail.com; 4 Institutes for Environmental Toxicology, Martin-Luther-Universität, Halle-Wittenberg and Faculty of Biomedical Engineering, Anhalt University of Applied Science, 06366 Köthen, Germany; sujiroshi@gmail.com; 5 Department of Oral and Maxillofacial Surgery, University Hospital Hamburg-Eppendorf, 20246 Hamburg, Germany; reiner.schnettler@mac.com (R.S.); m.barbeck@uke.de (M.B.)

**Keywords:** bone scaffold, additive manufacturing, 3D printing, bone regeneration, dentistry, bone augmentation

## Abstract

Three-dimensional (3D) printing has become an important tool in the field of tissue engineering and its further development will lead to completely new clinical possibilities. The ability to create tissue scaffolds with controllable characteristics, such as internal architecture, porosity, and interconnectivity make it highly desirable in comparison to conventional techniques, which lack a defined structure and repeatability between scaffolds. Furthermore, 3D printing allows for the production of scaffolds with patient-specific dimensions using computer-aided design. The availability of commercially available 3D printed permanent implants is on the rise; however, there are yet to be any commercially available biodegradable/bioresorbable devices. This review will compare the main 3D printing techniques of: stereolithography; selective laser sintering; powder bed inkjet printing and extrusion printing; for the fabrication of biodegradable/bioresorbable bone tissue scaffolds; and, discuss their potential for dental applications, specifically augmentation of the alveolar ridge.

## 1. Introduction

Additive manufacture (AM) has been shown as a promising fabrication technique for the production of bone replacement scaffolds. Customization and repeatability of designs, as well as the precise control over scaffold architecture, make AM superior to conventional techniques. The availability of additively manufactured permanent implantable medical devices is beginning to increase [1], however, there are yet to be commercially produced additively manufactured biodegradable/bioresorbable scaffolds.

This review will report on the research that was conducted into the AM of biodegradable/bioresorbable bone implants, broken down into the materials used. The end of the review will then be used to assess the suitability of each technique, based on the conducted research, for the application of alveolar ridge augmentation.

### 1.1. Alveolar Ridge Augmentation

Augmentation of the alveolar ridge is an important procedure for enabling the placement of dental implants, and thus, to restore both functionality and esthetic appearance. Successful implant placement is reliant upon adequate alveolar bone volume at the implant site to provide mechanical stability and anchor the implant in position. Augmentation can be achieved through the application of different bone augmentation materials. In this context, granular bone or bone blocks are applied to achieve bone augmentation via guided bone regeneration (GBR). The bone graft acts to provide mechanical support, space provision, blood clot stabilization, and bony ingrowth; these are the important factors that must be replicated in any AM design. 

**Figure 1:**
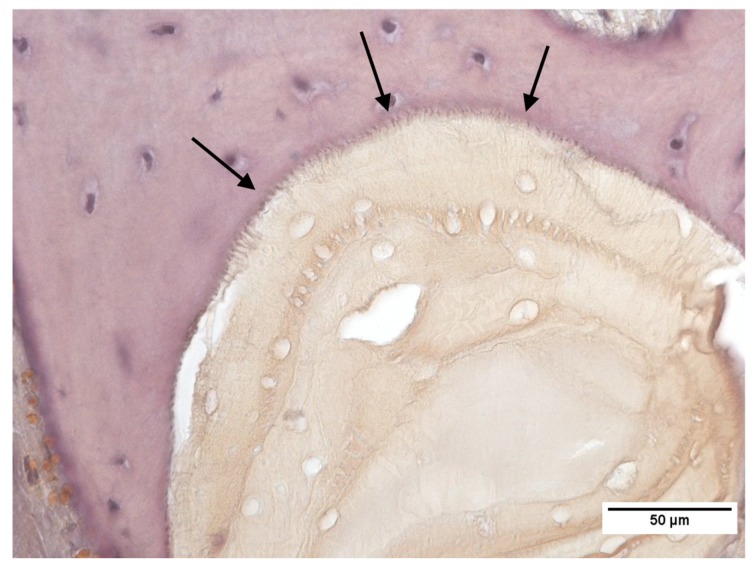
Xenogeneic bone graft (Cerabone^®^) showing seamless tissue integration. Arrows are used to highlight the bone graft/host bone tissue interface.

Bone grafting materials can be divided into autologous, allogenic, xenogeneic and alloplastic. Autografts, allografts and xenografts tend to be brittle due to their post-extraction processing, however demonstrate excellent tissue ingrowth [2,3], as demonstrated in Figure 1. Alloplastic scaffolds that are created through conventional manufacturing techniques tend to have poorly defined structures, due to a limited control of material placement during scaffold fabrication [4]. A common disadvantage for the currently used bone substitute materials is the variability and lack of reproducibility between individual bone grafts. 

### 1.2. Optimal Properties of Bone Tissue Scaffolds

Autografts are the gold standard in the regeneration of bone tissue, however due to donor’s site morbidity, low quality of geriatric/pathological sources, or the need for two invasive surgeries, alternative bone grafting scaffolds are needed. An idealized scaffold should be replaced by the host bone tissue, through osteoinductive and osteoconductive material properties [5,6]. The main types of degradable bone graft materials are natural polymers, synthetic polymers and bioceramics.

An idealized bone tissue scaffold for alveolar bone augmentations must fulfill a specific criteria: be biocompatible, match the physical properties of the bone tissue to be replaced, not to elicit a chronic immune response and to fully integrate with the bone [5,7]. The manufacturing process, as well as design parameters, such as porosity, pore size, scaffold interconnectivity, and mechanical strength, have been shown to influence the osteogenic cell interaction [8,9,10].

The mechanical properties of the scaffolds should be equivalent to that of the host bone. Alveolar bone has a cortical thickness between 2.1–2.4 mm and a density between 1.64–1.75 mg/cm^3^ [11]. Bone tissue scaffolds are usually compared to the compressive strengths of cancellous bone, which in the human mandible, ranges between 0.22 to 10.44 MPa with a mean value of 3.9 ± 2.7 MPa [12], however this can change depending on bone density, age, and gender [13,14]. Finite element analysis of dental implants during mastication has shown that, when experiencing an applied bite force of 146 N, a compressive stress of 62 MPa is produced on the surrounding alveolar bone that supports the dental implant [15]. Compressive forces on the alveolar bone increased to 122 MPa when the angular abutment of the implant was changed from 0° to 20°. This demonstrates the range of forces that the bone graft could be subjected to, and therefore, the necessity of the bone graft to fully integrate and be replaced by the host bone tissue, thereby increasing its mechanical strength to withstand such forces. Usually, after the placement of a bone graft and the insertion of a dental implant, there is a healing period to allow for tissue integration before the implant and the surrounding supporting structures must sustain mechanical loading.

Porosity, including pore size and interconnectivity, enable tissue penetration, surface area for biological fixation, and bony integration. Optimal osteoconductivity has been achieved with pore sizes similar to the native structure of cancellous bone [16,17,18], which has pore sizes ranging between 200–500 µm and a 30–90% porosity [19]. Even though small pore sizes in vitro induce earlier osteogenesis by limiting cell proliferation, larger pore sizes in vivo improve bone structure and tissue penetration [16]. 

**Figure 2:**
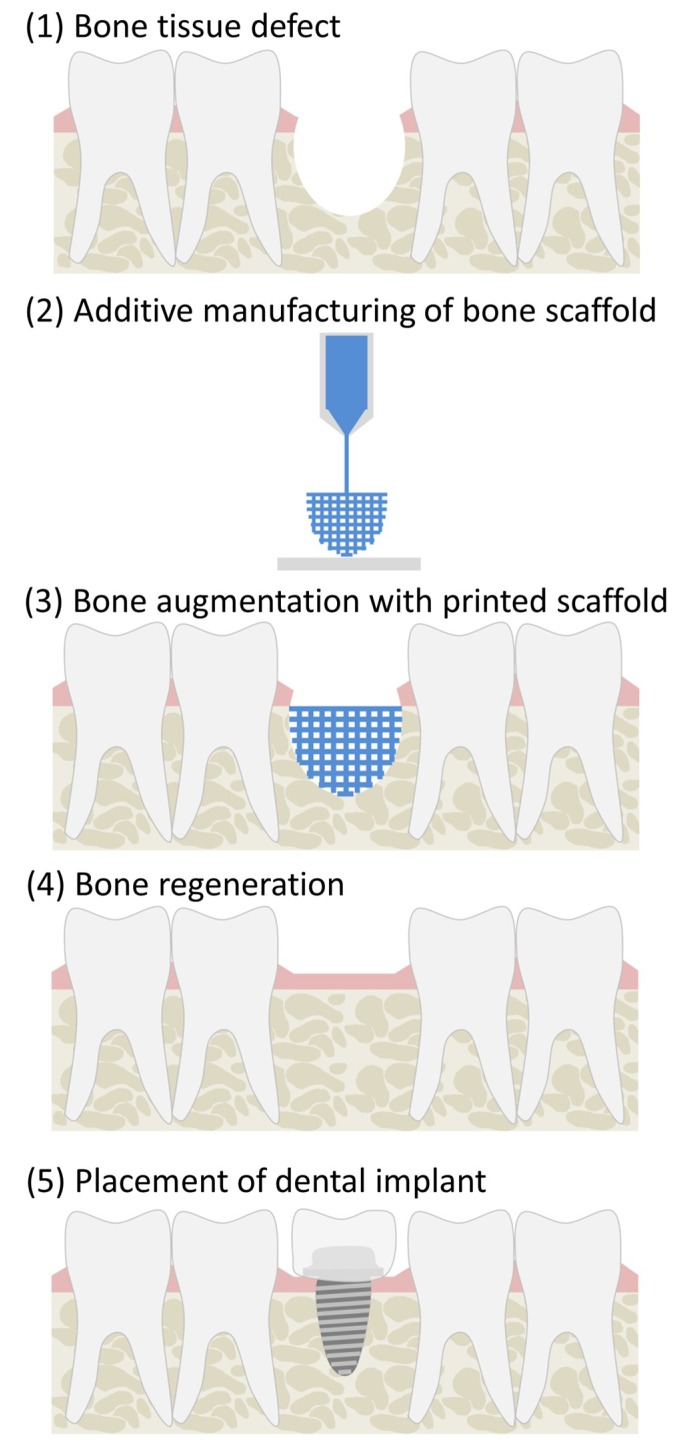
Alveolar ridge augmentation using an additively manufactured bone tissue scaffold. (**1**) a bone defect has formed in the alveolar ridge; (**2**) a bone scaffold is designed and then printed using additive manufacturing technology; (**3**) the printed bone scaffold is placed in the defect space to support bone regeneration; (**4**) new bone infiltrates the scaffold, eventually degrading or resorbing the structure; and, (**5**) a dental implant in positioned in the regenerated bone.

The ideal scaffold should be biodegradable/bioresorbable. Biodegradation and bioresorption are used to indicate the breakdown of a material when introduced into a living organism; however, these two terms have different meanings [20]. Biodegradation refers to the chemical breakdown mediated by agents, like cells, enzymes, or microorganisms. For example, natural polymers, such as collagen, which are broken down by collagenase enzymes, cleaving the molecular chain via its peptide bonds [21]. On the other hand, bioresorption implies that the implant and its by-products are removed by cellular activities, such as phagocytosis. An example of bioresorption would be osteoclasts remodeling implanted hydroxyapatite in vivo, where hydroxyapatite gets surrounded by a bony matrix and is slowly resorbed into the structure [22]. To produce a bone tissue scaffold with all the aforementioned characteristics, a variety of biodegradable/bioresorbable materials have been used along with different manufacturing techniques. Additive manufacturing offers the greatest possibilities for advancing the development of bone tissue scaffolds by providing control over scaffold design and properties, not attainable through current conventional methods [23]. Scaffolds can be designed on a computer and sent for manufacture, enabling the creation of complex, tailorable, and highly reproducible scaffolds. The process for using additively manufactured bone tissue scaffolds for repair of the alveolar ridge is depicted in Figure 2. As the materials being discussed in this review have an established biocompatibility and are biodegradable/bioresorbable, this review will focus on the other necessary properties that are required for successful bone augmentation achieved through the respective manufacturing technique. 

### 1.3. Additive Manufacturing Techniques (AM)

Three-dimensional (3D) printing, also known as additive manufacture, was developed in the 1980s and has since been applied to bone tissue engineering. In the present review, different additive manufacturing techniques are discussed. To control the precise porous architecture of a scaffold, various AM techniques have been developed. Advances in computer-aided design have brought rapid progress to fabricate custom-made scaffolds directly from computer data. In AM, scaffold architecture is manufactured by the processing of liquid or powder materials according to computerized designs. AM techniques have significant advantages over conventional fabrication techniques, as they can produce complex scaffolds with a precise external shape, internal morphology, and reproducible 3D architecture [24]. Among the different AM techniques, laser-based printing, such as stereolithography (SLA) and selective laser sintering (SLS), extrusion-based printing and inkjet printing are the most widely used 3D printing methods used for the fabrication of tissue scaffolds. Research conducted by each manufacturing technique for the production on bone tissue scaffolds will be discussed and sub-divided by the main biodegradable/bioresorbable material used.

## 2. Stereolithography

**Figure 3:**
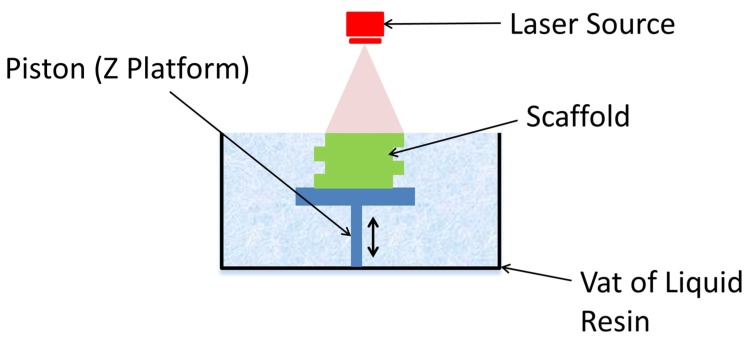
Schematic of stereolithography (SLA) printing process. The laser source cures the top of the liquid resin in a predetermined pattern. The platform is then lowered by the height of the cured resin and the process is repeated.

Stereolithography (SLA) uses an ultraviolet laser to cure liquid resin into a hardened material in a layer-by-layer fashion. It consists of a reservoir that contains a photosensitive polymer resin and a moveable build platform. This technique works by focusing a UV laser on to the photopolymer resin [25], as demonstrated in Figure 3. Printing often requires the use of photoinitiators to help with the curing process, however these can have toxic effects on the cells if any residue remains in the parts [26]. SLA has one of the highest resolutions; 5–300 µm [26], accuracies and the smoothest surface finish of all plastic 3D printing technologies, but the main benefit of SLA lies in its versatility. SLA is a great option for highly detailed scaffolds, requiring tight tolerances and smooth surfaces, such as molds, patterns, and functional parts, as it can achieve a spatial resolution of approximately 50 μm [25]. Various techniques that fall under SLA, include: two-photon polymerization (2PP) [25], holography [27], and visible light-based [28] techniques. The various SLA techniques offer different possibilities, such as improved resolutions of up to 200 nm with 2PP or the use of visible light to avoid the negative impact of UV light on cells. A wide range of materials can be prepared for SLA (compared in Table 1), however they are limited by their ability to be processed into a photo-crosslinkable hydrogel, modified by the addition of photo-crosslinkable groups along the molecular chain [29,30,31].

### 2.1. Natural Polymers

#### 2.1.1. Gelatin

Gelatin methacrylate (GelMA) is the product of combining methacrylate groups with amine-containing side groups of gelatins, and it becomes photo-crosslinkable in the presence of a photoinitiator [30]. Scaffolds that are made from GelMA have high levels of cell proliferation and migration in two and three-dimensional cultures [31]. Benton et al. investigated the basic mechanism of photo-crosslinking material through SLA, by curing two-dimensional (2D) layers of GelMA with UV exposure and different concentrations of photoinitiator. The photoinitiator concentrations affected porosity in the GelMA by polymerization-induced phase separation [31]. A hydrogel of GelMA was mixed with different concentrations of photoinitiator, pipetted between two glass slides and exposed to UV radiation. The concentration of photoinitiator affected the degree of polymerization, and thereby affecting how much of the structure gelled or remained liquid. As the degree of polymerization varied, so did the pore size, whereby 0.5 wt % Irgacure 2959 produced pores with an area of 1800 µm^2^ and a 0.05 wt % produced pores with an area of 400 µm^2^ [31]. This can be transferred to scaffold manufacture, where researchers may want to increase material porosity via other means than the scaffold design. 

An advantage of GelMAs is that they are low cost and easy to produce [29]. However, a fast degradation rate (complete mass loss in <90 h when submerged in collagenase [31]) and poor mechanical properties (compressive strength between 5–30 kPa [29]) may make it unsuitable for some applications in hard tissue regeneration.

#### 2.1.2. Silk Fibroin 

Silk fibroin (SF) is a versatile natural polymer that can be easily adapted for additive manufacture [41,42]. Silk methacrylate (SilMA) scaffolds were prepared by the addition of methacrylate groups to the amine-containing side groups of silk [32]. Mechanical testing showed compressive strengths of 910 kPa, which are 30 times greater than GelMA samples. Cell proliferation rates were similar for both SilMA and GelMA samples. Due to the improved mechanical properties and similar cell proliferation, SilMa scaffolds could be more suitable for bone tissue scaffold fabrication.

Silk Fibroin/GelMA composite scaffolds that were developed by W. Xiao et al. had a high mechanical strength and also the ability to physically crosslink using UV radiation and methanol [30]. It was hypothesized that only GelMA would crosslink when exposed to UV light, immobilizing the amorphous SF. After printing, methanol treatment could be used to induce crystallization of the SF and introduce more physical crosslinking sites. Without the methanol treatment, the compressive strengths of all samples remained close to the compressive strength of GelMA, <5 kPa, however with a methanol treatment, compressive strengths reached 75 kPa (2 wt % SF). The inclusion of SF also caused a reduction in degradation rate, where after 72 h in degradation media, GelMA scaffolds had lost 60% of their mass whilst GelMA-SF scaffolds had lost less than 5%. Cell studies demonstrated lower proliferation rates with increasing SF concentrations, possibly due to β-sheets configuration (a crystalline form of SF), not being favorable for cell adhesion and proliferation. Overall, GelMA-SF constructs offer greater suitability for bone scaffolds in comparison to pure GelMA.

#### 2.1.3. Chitosan

Chitosan and polyethylene glycol diacrylate (PEGDA) photo-crosslinkable blends were developed by Morris et al. [33]. PEGDA exhibits neither cytotoxicity nor immunogenicity and it has great aqueous solubility; however, PEGDA is bioinert and it suffers from low receptiveness to cell adhesion [43]. Cells seeded onto pure PEGDA surfaces remained spherically shaped and it had a reduced cell viability due to poor cell anchorage to its bioinert surface [33,44,45]. Increasing chitosan concentration improved cell viability but reduced mechanical properties. Pure PEGDA scaffolds had a compressive modulus of 1125 ± 68.05 kPa, which dropped to <200 kPa for samples with the highest chitosan concentration (1:5, chitosan to PEGDA). 

#### 2.1.4. Alginate

Generally, different concentrations of photoinitiator will affect material mechanical properties. Using alginate methacrylate, elastic moduli of 3.3 kPa (compliant), 7.9 kPa (moderate), and 12.4 kPa (stiff) were obtained by using different concentrations of photoinitiator [36]. Moderate to stiff alginate gels exhibited better cell viability rates after seven days in comparison with complaint gels, possibly linked to the adhesion of the cells. Jeon et al. studied the different degrees of methacrylation on alginate degradation kinetics and mechanical properties [46]. The highest degree of methacrylation resulted in the highest elastic modulus of 174.77 ± 14.88 kPa and the lowest degradation rate, although were completely degraded within five weeks in vitro. 8% methacrylate concentration exhibited a fast degradation rate, whereby after one week the samples had lost 95.67% of their original mass. Indirect cell culture of chondrocytes was performed to evaluate cytotoxicity. Cells exposed to the alginate hydrogel with highest methacrylate concentration (25%) exhibited a slightly lower viability compared to a control of no scaffold.

### 2.2. Synthetic Polymers

#### 2.2.1. Poly(Propylene Fumarate) (PPF)

Poly(propylene fumarate) (PPF) was an early choice of material for SLA bone tissue scaffolds [47], and offers excellent cellular adhesion [48]. Lee et al. fabricated PPF scaffolds using a PPF/diethyl fumarate photopolymer that was embedded with poly(dl-lactic-co-glycolic acid) (PLGA) microspheres loaded with bone morphogenetic proteins [49]. When combined with the photoinitiator bisacrylphosphrine oxide and exposed to a UV light, the PPF will form a crosslinked polymer network [50]. Cell viability was tested in vitro using pre-osteoblast MC3T3-E1 cells, seeded on SLA scaffolds, and scaffolds fabricated using a conventional technique of solvent casting and particulate leaching. Porosities of the scaffolds were 69.9% and 76.6%, respectively. Over a two-week period, the SLA scaffolds showed enhanced cell attachment and proliferation rates as compared to the conventionally fabricated scaffolds. The BMP-loaded scaffolds produced the highest alkaline phosphatase (ALP) activity and Collagen I expression when compared to scaffolds that were not loaded with microspheres. For in vivo assessment, BMP-loaded and unloaded SLA scaffolds, and conventional scaffolds were implanted in calvarial defects in Wistar rats. After four weeks, the defect size was reduced in all groups except for a negative control. After 11 weeks, BMP-loaded SLA scaffolds resulted in the highest percentage of new bone tissue formation (80.9 ± 5.09%), followed by unloaded SLA scaffolds (32.85 ± 8.27%), and finally conventionally fabricated scaffolds (15.15 ± 3.00%).

#### 2.2.2. Polycaprolactone (PCL)

Methacrylated PCL scaffolds fabricated by Elomaa et al. had a tensile strength between 2.02 ± 2.87 MPa [37]. Tissue culture studies showed that the PCL scaffolds exhibited a metabolic activity similar to the control group (tissue culture polystyrene). PCL incorporated with bioactive Glass S53P4 (BG) have improved mechanical properties in both dry and wet conditions [34]. Samples containing 20 wt % BG had compressive strengths of 3.4 and 2.5 MPa, in dry and wet conditions, respectively. An increasing BG content improved the compressive strength and cell activity of the scaffolds.

#### 2.2.3. Polylactic Acid (PLA)

Methacrylated PLA scaffolds were incorporated with different concentrations of HA and Triethylene glycol dimethacrylate (TEGMA) to improve the mechanical strength [39]. It was shown that an intermediate concentration of HA provided the best flexural strength (9.43 ± 3.2 MPa for 40 wt % HA) [39].

Methacrylated poly (d,l-lactide) (PDLLA) scaffolds that were prepared using the photoinitiator Irgacure 2959 had a flexural strength between 80–97 MPa [51]. When fabricated using a fumaric acid monoethyl ester-functionalized PDLLA (3-FAME PDLLA), scaffolds had a tensile strength of 1.3 MPa. With the addition of *N*-vinyl-2-pyrrolidone (NVP), the tensile strength increased to 56 ± 10 MPa. In vitro tests suggest that the mechanical properties and water uptake can be tailored by changing the NVP content without any negative consequences on the cell adhesion properties, and therefore can successfully be used for improving the mechanical properties of PDLLA in situ [52]. 

#### 2.2.4. Poly(trimethylene Carbonate) (PTMC)

Compared to other polyesters, PTMC is advantageous in the kinetics and mechanism of its resorption. Polyester scaffolds undergo bulk degradation through hydrolysis that can cause an inflammatory reaction due to the local accumulation of acidic oligomers. PTMC, however, is degraded by surface erosion, enabling it to maintain mechanical integrity and does not degrade into acidic by-products. Methacrylated PTMC (PTMC-MA) and PTMC-MA/nHA scaffolds fabricated using SLA were tested in vitro for cell proliferation and osteogenic capacity [40]. Proliferation rate and colonization were observed in both samples, but were lower for samples containing HA. Osteogenic differentiation of cells on the PTMC-MA/HA exhibited higher collagenous extra cellular matrix secretion, ALP activity, and calcium deposition. Implanted into calvarial defects in rabbits and compared to sham surgeries, the PTMC-MA/HA scaffolds produced higher bone volumes, with 60% of pores being filled with new bone. Distance osteogenesis was observed to occur at the sham surgery sites, while contact osteogenesis occurred for the PTMC-MA/HA scaffolds.

#### 2.2.5. Poly(ethylene Glycol) Divinyl Ether (PEG-DVE)

As an alternative to the use of photoinitiators, research has been conducted investigating thiol-ene reactions to produce photopolymer networks [38,53,54]. A thiol-ene reaction occurs between alkene- and thiol-monomers to form an alkyl sulfide group, regarded as a photo-triggered click chemistry reaction, which can be utilized for initiator-free photopolymerization [54]. Different ratios of thiol, pentaerythritol tetrakis(3-mercaptopropionate) (PETMP), and alkene, poly(ethylene glycol) divinyl ether (PEG-DVE), (1:1 and 2:1, thiol:alkene) were compared for their biocompatibility [38]. A 1:1 ratio demonstrated higher cell viability over three days, as increased thiol content caused higher cytotoxicity. Although crosslinking occurred without the presence of a photoinitiator, cytotoxic effects were still present. 

#### 2.2.6. 2-Ethylhexyl Acrylates (EHA)/Isobornyl Acrylate (IBOA)

A. Malayeri et al. developed a hierarchically porous polymer scaffold, known as a polyHIPE due to its synthesis using high internal phase emulsions (HIPEs), using 2-ethylhexyl acrylates (EHA) and isobornyl acrylate (IBOA) [55]. PolyHIPEs are a class of material where porosity is created via a phase-separation process, caused by mixing two immiscible liquids in the presence of an emulsifier [56]. 

Both EHA and IBOA are immiscible with water. Both EHA and IBOA were mixed together and were combined with photoinitiators (diphenyl(2,4,6-trimethylbenzoyl) phosphine oxide/2-hydroxy-2-methylpropiophenone), after which, water was slowly added to the solution. Using an SLA laser to photopolymerize the solution effectively cured a material that already had a porous structure. Advantages to this approach include the possibility to create controllable hierarchical porosity (macro- and microporosity), as well as the feasibility of adding nanoparticles to the structure, e.g., hydroxyapatite particles. Fabricated 3D structures exhibited porosity on both macro and micro levels using this reaction. Human osteosarcoma cells (MG-63) that were seeded onto the scaffolds had slightly lower viabilities than cells in the tissue culture plastic control; however, the MG-63 cells seeded on the polyHIPEs developed tumor spheroids, suggesting that polyHIPEs are a better biomimetic structure of trabecular bone. 

### 2.3. Bioceramics

#### Tricalcium Phosphate/Hydroxyapatite

Bioceramic and bioceramic/PCL scaffolds were designed and fabricated by Seol et al. [35]. Ceramics are not photocurable and they require a photocurable resin to bind the particles together, in this instance, HA and TCP (7:3 wt %). The bioceramics slurry was mixed with a photocurable resin (FA1260T; SKCytec, Seoul, Korea), at a 20% volume ratio of bioceramics to resin. After curing with SLA and the removal of the uncured solution, the scaffolds were sintered at 1400 °C to remove the solidified photocurable resin and fuse the bioceramic particles together. For bioceramic/polymer scaffolds, PCL solution was used to infiltrate the scaffolds after sintering. The average compressive strengths were 2.04 ± 0.12 MPa for pure bioceramic and 4.55 ± 0.21 MPa for bioceramic/PCL. Cell viability of the bioceramic/polymer scaffolds was less than pure bioceramic scaffolds. However, bioceramic/PCL scaffolds exhibited a higher expression of osteogenic markers, both in normal and osteogenic mediums.

## 3. Selective Laser Sintering

**Figure 4:**
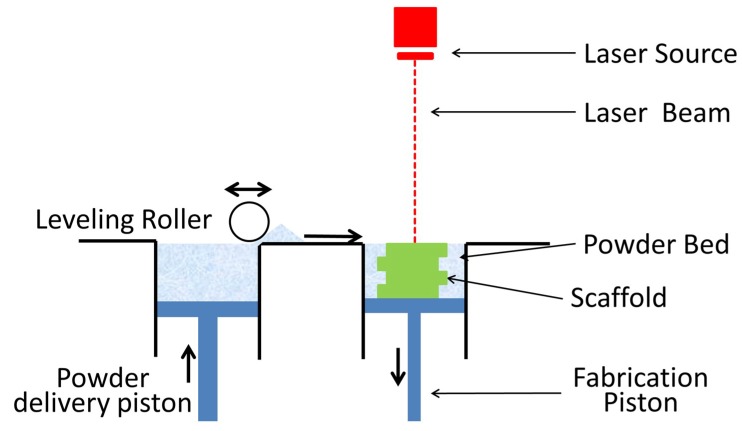
Schematic of selective laser sintering (SLS) process. A laser source sinters/melts the top layer of powder in a powder bed in a predetermined pattern. The powder bed is lowered in height and a fresh layer of powder is positioned on top via a leveling roller. The process is then repeated.

Selective laser sintering (SLS) uses a high-powered carbon dioxide laser to fuse small particles of polymer powder, as depicted in Figure 4. Most commonly used materials in SLS are the polymer PCL, calcium phosphates, or composites of polymer and bioceramic [57]. Fabrication of ceramics using SLS is generally considered difficult due to the fast heating and cooling rates that are induced by the high temperature laser, which produces scaffolds that are usually fragile [58,59]. The main advantage of SLS is its capability of producing highly detailed prints with thin walls [60]. However, in comparison to the other AM techniques, it has a poor dimensional accuracy of just 150–180 µm [57]. Other issues that are associated with SLS include the inability to incorporate growth factors and cells during printing [57], as well as shrinking and warping of the scaffold due to thermal distortion [58]. A variety of materials can be used for SLS (summarized in Table 2), however natural polymers cannot be utilized in this technique because of the high temperatures that are generated by the laser. 

### 3.1. Bioceramics

#### 3.1.1. Magnesium Silicate

Magnesium silicate (Mg_2_SiO_4_), also known as forsterite, is a bioceramic that exhibits good biocompatibility and mechanical strength, however it suffers from poor bioactivity and a slow degradation rate. To improve its degradative and bioactive properties, Sun et al., combined forsterite with a calcium inosilicate, wollastonite (CaSiO_3_) [61]. Using SLS with a laser power and spot diameter of 8.5 W and 1 mm, scaffolds were fabricated that had a compressive strength of 29.81 MPa with 0 wt % wollastonite, which increased to 40.29 ± 1.32 MPa with the inclusion of 20 wt % wollastonite. Faster degradation and cell proliferation rates were observed with scaffolds containing 20 wt % CaSiO_3_.

#### 3.1.2. Tricalcium Phosphate (TCP)

In vitro studies have shown great biocompatibility and proper degradation of both HA [68] and β-TCP [69] SLS fabricated scaffolds. β-TCP is mostly used in low to non-load-bearing applications due to its brittle nature. The addition of oxide-based dopant increases the mechanical strength and lowers its degradation rate [62]. In a study that was conducted by Feng et al., β-TCP was doped with different proportions of Zinc oxide, as Zn has a proliferative effect with osteoblastic cells and an inhibitive effect with osteoclastic cells [62]. Scaffolds that were fabricated using a laser power and spot diameter of 12 W and 1.2 mm, increased in compressive strength from 3.01 MPa for pure β-TCP to 17.89 MPa for TCP with 2.5 wt % ZnO. Scaffolds with 2.5 wt % ZnO exhibited optimal mechanical strength and biocompatibility when compared to other concentrations.

#### 3.1.3. Hydroxyapatite (HA)

Another study by Shuai et al. was conducted to examine the effects of HA incorporated with β-TCP via SLS. Fracture toughness of HA and β-TCP are 0.83 and 0.98 MPa, respectively [63]. A maximum fracture toughness of 1.33 MPa and compressive strength of 18.35 MPa were recorded with the ratio of 30:70 β-TCP/HA, however better degradation kinetics were observed for scaffolds with a higher β-TCP content [63].

### 3.2. Polymers

#### 3.2.1. Polycaprolactone (PCL)

PCL is commonly used in SLS because of its low melting (59–64 °C) and glass-transition temperatures (–60 °C) that make it easily processable [70]. Scaffolds were fabricated using a laser power and spot diameter of 4.1 W and 450 µm. Ultimate compressive strengths were dependent on scaffold design, where the highest compressive strength reported was 10 ± 0.62 MPa for a scaffold porosity of >50% [71]. Another PCL scaffold with a compressive strength of 2.3 MPa and 50% porosity was implanted into minipigs. Bone ingrowth and cartilage ingrowth on the articular surface were observed and after 3 months the defects had completely healed [64].

Combined with bioceramic particles, SLS fabricated PCL scaffolds show further potential for bone tissue applications [60,72,73,74]. In vivo implantation with 15 wt % nHA, developed greater bone ingrowth in comparison to pure PCL and a sham surgery [66]. The inclusion of β-TCP reportedly increased the mechanical strength of PCL scaffolds, increasing the modulus from 2.0 to 3.0 MPa, however in vivo results showed that in comparison to a pure β-TCP scaffold, there was less bony ingrowth [65].

#### 3.2.2. Poly(hydroxybutyrate) (PHB)

Poly(hydroxybutyrate-co-hydroxyvalerate) (PHBV) scaffolds and PHBV/TCP/HA scaffolds were fabricated using SLS, and a laser power of 14 W for pure PHBV scaffolds and 15 W for PHBV/TCP/HA. Compressive strength for the PHBV scaffolds increased from 0.475 to 0.55 MPa with the incorporation of TCP/HA. Both scaffolds exhibited good cell viability and ALP activity, with slightly better results being recorded with the incorporation of TCP/HA nanoparticles [75].

## 4. Powder Bed Inkjet Printing

**Figure 5:**
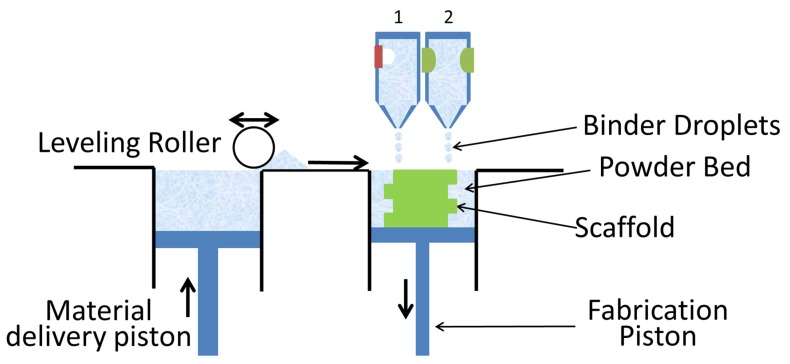
Schematic of Two Different Inkjet Printing Mechanisms over a powder bed: 1. Thermal-based, 2. Piezoelectric-based. The inkjet printheads dispense a binding solution to the powder bed below. The powder bed is lowered in height and a fresh layer of powder is positioned on top via a leveling roller. The process is then repeated.

In powder bed inkjet printing, droplets of dilute solutions or biomaterials are dispensed, driven either by thermal or piezoelectric processes into a powder bed. The prinited ink acts as a binder solution to a bulk material positioned within the powder bed [76,77], as shown in Figure 5. Thermal printing processes create a localized temperature between 100 to 300 °C to nucleate a bubble and eject droplets [78,79]. Disadvantages of using thermal inkjet printing include the effect of shear and thermal stresses on natural polymer inks, as well as an inconsistent droplet volume [69,71]. In piezoelectric technology, drops are generated by pressure or accoustic waves that are produced via a piezoelectric actuator [24,78]. Advantages of piezo inkjet printing include its low cost and the ability to print a large variety of materials with the choice of polar and non-polar solvents. The main disadvantage is a requirement for low concentration inks, as high viscosities, which are caused by concentrated inks, dissipate accoustic and pressure waves before a droplet can be ejected [80].

The use of powder bed inkjet printing in the production of bone scaffolds is advantageous due to the variety of materials that can be used, limited only in that the material must be in a powder form. Bone grafts manufactured with this technique have shown promising results, ranging from their mechanical properties (Table 3) to a successful clinical trial [81,82,83].

### 4.1. Bioceramics

#### 4.1.1. Hydroxyapatite

Powder printed pure HA scaffolds have been shown to offer excellent biocompatibility [94], however are usually associated with a brittle structure. To improve the brittle nature of HA structures, Stevanovic et al. used polymer infiltration after sintering [95]. The mechanical properties were improved as the polymer bridges any cracks that form in the HA scaffold when the structure is under load. The HA scaffolds were initially fabricated using an acidic solution of phosphoric acid (15 wt %) and citric acid (10 wt %), which were then sintered for 1 h at 1425 °C. The scaffolds were then infiltrated with either gelatin, PCL or PVA. However, each layer of applied polymer reduced scaffold porosity, ranging from 2% reduction for PCL, up to 60% for gelatin. Polymer infiltration improved compressive strengths from 0.8 MPa, up to 3.7 MPa, 1.03 MPa, and 0.9 MPa after the application of gelatin, PVA, or PCL, respectively.

Another way of improving the mechanical properties of HA scaffolds has been to use a distribution of different particle sizes. Using an optimal particle size ratio increases the total surface contact between neighboring bioceramic particles, thereby improving the fusion of the particles during sintering. Using powder bed inkjet printing and a binder that is composed of 20 wt % dextrin and 2.5 wt % saccharose, to produce scaffolds that were then sintered at 1250 °C; a bimodal powder with a large particle size between 63–89 µm contributing to >75 wt % gave the best compressive strength (13.7 MPa) [96].

**Figure 6:**
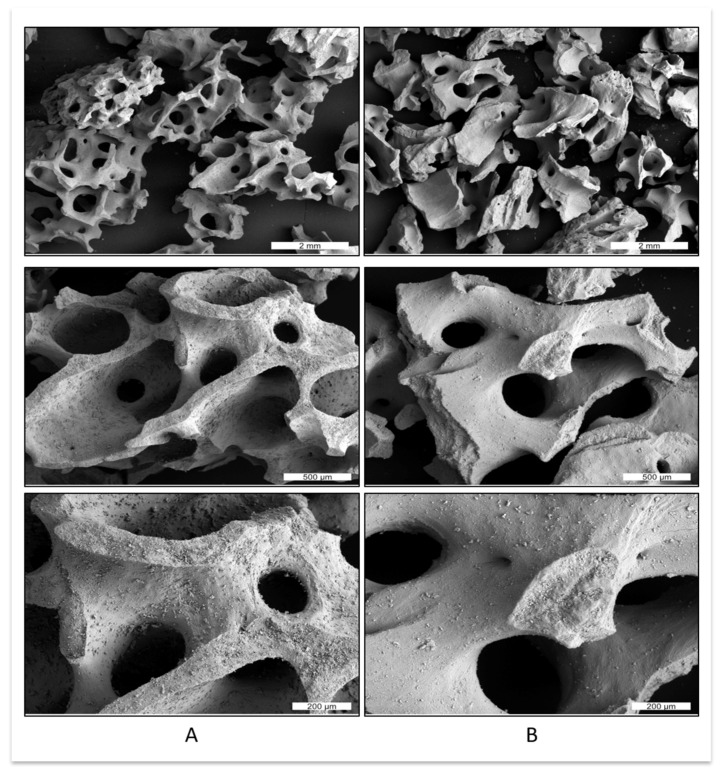
Comparison of two different xenogeneic bone graft materials: (**A**) sintered xenograft (Cerabone^®^) and (**B**) non-sintered xenograft (BioOss^®^). For example, xenograft (**A**) has a rougher surface in comparison to xenograft (**B**).

Polymeric binders can be used to bind the ceramic particles together. Seitz et al. produced HA scaffolds using a polymeric binder Schelofix (Friedrich-Baur-Institut, Bayreuth, Germany) [94]. The scaffolds were sintered at 1250 °C, pyrolyzing the polymeric binder, resulting in a compressive strength of 21.2 ± 2.2 MPa. The biocompatibility of HA and TCP scaffolds that were created using the same technique were evaluated by Warnke et al. [97]. Seeded with osteoblasts, both HA and TCP samples had higher cell proliferation rates after seven days in cell culture when compared to BioOss^®^, a deproteinized bovine bone mineral. HA scaffolds exhibited the highest biocompatibility and lowest cytotoxicity compared to the TCP scaffolds and the deproteinized bovine bone mineral. It is generally accepted that xenogeneic material produces better results in vivo than alloplastic [98], however, the different processing of the xenogeneic material can change the material properties [99], as demonstrated in Figure 6.

HA samples implanted into calvarial bone defects in rabbits, were designed either with channels or as a porous structure [100]. After eight weeks, the HA scaffolds contained new bone and soft tissue in their macro and microscopic pores and vascularization along the channels or porous network. When compared to an autograft, the porous HA scaffold produced the most similar results.

#### 4.1.2. Tetracalcium Phosphate (TTCP)

Tetracalcium phosphate (Ca_4_(PO_4_)_2_O) (TTCP) is more reactive than other calcium phosphates (CPs), and it is a common component of bone cements. TTCP scaffolds with a macro porosity of 39.4 ± 1.5%, had a compressive strength of 1.3 ± 0.2 MPa after printing that increased to 4.0 ± 0.4 MPa after an immersion treatment in phytic acid [89]. An immersion treatment in phytic acid enabled the formation of harder calcium phases, such as calcium phylate, which are mechanically strong, but are also more easily degraded and resorbed in comparison to HA. The mechanical properties of the printed scaffolds were analyzed as the TTCP degraded in a PBS solution. After seven, 15, and 28 days, the compressive strengths dropped to 3.6 ± 0.5 MPa, 3.1 ± 0.5 MPa, and2.8 ± 0.2 MPa, respectively [89].

#### 4.1.3. Dicalcium Phosphate (DCP)

Scaffolds printed without a subsequent heat treatment were produced from either brushite (DCPD) or HA. By not including a heat treatment, vascular endothelial growth factor (VEGF) and copper(II) ions could be included [84]. The scaffolds had a compressive strength of 22.3 ± 1.5 MPa (DCPD) and 5.8 ± 0.3 MPa (HA). After 15 days implanted into the peritoneal cavity in mice, implants with angiogenic inclusions had developed a vascular network spanning the device, as compared to a 2 mm penetration for scaffolds without inclusions. The angiogenic scaffolds had a micro vessel network lined with endothelial cells with vessels aligned parallel to pore direction.

Two of the most commonly used DCPs are brushite and monetite. The mechanical properties of brushite scaffolds are slightly better than that of monetite scaffolds. In a study that was performed by Klammert et al., brushite scaffolds that were fabricated by printing 20 wt % phosphoric acid onto TCP powder had a compressive strength of 23.4 ± 3.3 MPa compared to 15.3 ± 1.1 MPa for printed monetite scaffolds [90]. Seeded with osteoblastic cell line MC3T3-E1 cells, brushite demonstrated higher cell viabilities and ALP activity than the monetite samples, however in comparison to titanium positive controls, both CP scaffolds gave significantly lower results.

Brushite and monetite cranial and maxillofacial implants designed to fit a cadaver skull using CT data were shown to have a high degree of accuracy of fit [101]. The bending strength of the brushite scaffolds was 5.2 ± 0.8 MPa with a porosity of 28.2%, whilst the monetite scaffolds had a bending strength of 3.9 ± 0.5 MPa and a porosity of 34.6%. Due to its mechanical and biocompatibility properties, brushite may offer preferential properties for bone tissue engineering than monetite.

Monetite bone block onlay grafts with a 44% porosity and a 15 MPa compressive strength were implanted into the cranium of rabbits and compared to autologous bone onlay grafts [91]. Histological analysis that was performed at eight weeks showed that the printed monetite blocks had completely integrated with the native bone via its resorption and subsequent replacement with bone tissue and showed signs of calcification similar to that of the calvarial bone. Overall, the printed implants produced comparable results to that of autologous bone.

#### 4.1.4. Tricalcium Phosphate (TCP)

Concentrations of chemical binders have been shown to control the intensity of bonding reactions between TCP particles. Gbureck et al. found that increasing the concentration of the phosphoric acid binder from 5% to 30% improved the mechanical properties of TCP scaffolds from 0.9 to 8.7 MPa, respectively [87]. The type of heat treatment can also change the material properties. Microwave sintering of TCP scaffolds causes increased shrinkage and therefore densification [102]. A microwave sintered TCP scaffold with 500 µm designed pore sizes had a maximum compressive strength of 10.95 ± 1.28 MPa as compared to 6.62 ± 0.67 MPa for a standard sintered TCP scaffold.

CT data can be used to improve scaffold designs and create a better fit to the defect dimensions. α-TCP cranial plates designed using CT data and printed using a binder solution of 5% sodium chondroitin sulfate and 12% disodium succinate, were implanted into beagle dogs [85]. Blocks printed of the α-TCP that had a 61% porosity and had a compressive strength of 18.6 MPa. There was improved bone growth for the printed α-TCP samples compared to a standard HA bone block; however, both implants were predominantly infiltrated by connective tissues. Poor bone growth into the HA implant was linked to a reduced contact between the HA implant and the native bone. The HA bone block had to be cut into shape, whereas the 3D printed scaffolds were printed to match the defect dimensions.

Doping the TCP scaffold with bioactive compounds can improve biocompatibility [103]. To facilitate bone formation and faster mineralization, TCP scaffolds were doped with SrO and MgO [104]. Doping TCP with Sr and Mg ions densified the scaffolds due to substitutions within the TCP lattice. Doped TCP scaffolds with a pore size of 1000 µm achieved a compressive strength of 12.01 ± 1.56 MPa as compared to 10.95 ± 1.28 MPa for pure TCP. The doped TCP scaffolds achieved faster bone formation and developed similarities to the native tissue when implanted into rat distal femoral defects.

TCP scaffolds doped with silica (0.5 wt %) and zinc oxide (0.25 wt %) retarded the transition of β to α phase during sintering above 1150 °C, resulting in significantly higher compressive strengths [105]. Doped samples had a porosity of 41.04% and 27.26% and almost double the compressive strengths of pure TCP samples. Proliferation rates of human fetal osteoblastic (hFOB) cells seeded on the doped TCP scaffolds were significantly higher than that for pure TCP scaffolds.

#### 4.1.5. Octacalcium Phosphate (OCP)

Octacalcium phosphate scaffolds can be produced using low temperature printing and they have better mechanical properties as compared to TCP. Komlev et al. made green TCP cranial plates by printing a 1% phosphoric acid binder onto a TCP powder, which resulted in a compressive strength of 2.5 MPa [86]. The formation of octacalcium phosphate was induced by immersing the TCP scaffolds in ammonium phosphate solution, followed by sodium acetate solution. The octacalcium phosphate cranial plates had a compressive strength around 7.5 MPa. After implantation into rabbits, bone tissue integrated along the edges of the implant as well as along the internal and external surfaces. However, within the central region of the plate, no osteogenesis had occurred and fibrous tissue had penetrated the pores.

#### 4.1.6. Calcium Polyphosphate (CPP)

Printed CPP scaffolds have shown isotropic effects due to print orientation. Shanjani et al. found that thescaffolds were 48% stronger when printed layers were stacked parallel to the applied compressive loads, increasing from 33.86 ± 6.32 MPa to 50.17 ± 4.74 MPa [106]. The isotropic effects were attributed to the orientation of the powder bed particles. During the application of powder between printed layers, the roller action could cause the non-spherical particles to orientate with their larger facets parallel to the build plane. During sintering it is via the large facets that the most predominant interparticle connections are made, hence providing isotropic properties.

To compare the in vivo response of the printed CPP scaffolds in comparison to conventionally made CP scaffolds, Shanjani et al. fabricated scaffolds using the above mentioned method, as well as by a conventional technique. Both methods produced scaffolds that had a ~30% porosity, and were implanted into the medial femoral condyle of rabbits over a six-week period [107]. All scaffolds demonstrated a uniform bone ingrowth and a degradation rate between 7–9%. The tissue response did not seem to be influenced by any isotropic effects that are caused by the print orientation. Overall, the study indicated that a similar in vivo response for both fabrication methods and therefore the suitability of powder bed inkjet printing for the fabrication of CPP bone tissue scaffolds.

#### 4.1.7. Biphasic Calcium Phosphate (BCP)

BCP composition will influence scaffold mechanical and biocompatibility properties, whereby an optimum 60:40 ratio of HA to β-TCP has been reported to give the highest cell proliferation rates whilst retaining good mechanical properties [88]. In a preclinical study, it has been shown that the 60:40 biphasic composition provides excellent degradation kinetics, combining the characteristics of both HA and β-TCP compounds [108].

The type of binder used will influence the mechanical properties of the scaffold. Wang et al. found that BCP scaffolds held together with PVA, which acted as a physical binder between particles, were mechanically weaker than a phosphoric acid binder, which chemically bonded the particles together [88]. PVA binder samples had compressive strengths between 1.21–1.54 MPa, a Young’s modulus between 38–70 MPa, and a strain to failure range between 2.5–3.4%, whilst the phosphoric acid binder samples had a compressive strength range of 2.36–2.81 MPa, a Young’s modulus between 100–147 MPa, and a strain to failure between 3.0–2.8%.

Inzana et al. used a low temperature fabrication of TCP scaffolds using a binder of a phosphoric acid solution containing collagen [57]. Phosphoric acid concentrations ≥8.75 wt % caused the collagen to denature. Lower phosphoric acid concentrated solutions containing 1 wt % collagen produced a similar mineral density, mineral content, and net mineralized volume when compared to an autograft when implanted into mid-femoral defects in mice. However, after nine weeks, neither the scaffold nor the autograft had a torsional strength that is comparable to an intact murine femur (19.4 ± 5.6 N mm).

#### 4.1.8. Calcium Sulfate (CS)

The temperature used during post printing heat treatments has been shown to affect the mechanical and biocompatible properties of calcium sulfate scaffolds [92]. Heated to ≥1150 °C, CS scaffolds were shown to have the highest compressive strength (2.47 MPa) and Young’s modulus (52.11 MPa). Scaffolds that were heat treated at 300 °C exhibited sever toxicity (below 60% viability) due to remnants of the binder. Samples heat treated between 500–1000 °C were non-cytotoxic due to pyrogenesis of the binder at 500 °C, however these scaffolds had poor mechanical properties due to insufficient densification. Overall, samples receiving a heat treatment of 1000 °C and above had the best mechanical properties and were the least cytotoxic. A porous scaffold that was sintered at 1250 °C had the most idealized properties with a compressive strength of 0.55 MPa and a Young’s modulus of 58.12 MPa.

Farzadi et al. found that layer thickness and print orientation were also important for mechanical properties of non-heat-treated scaffolds [80]. A peak compressive strength (~0.5 MPa) and Young’s modulus (~30 MPa) was measured for Scaffolds printed with a *Z* axis orientation, which was significantly higher than that of scaffolds that were printed in an *X* or *Y* orientation. Increasing the print delay time also had an effect. Prolonging the print delay time from 50 to 300 ms increased the compressive strengths from ~0.4 to ~0.8 MPa and Young’s modulus from ~0.6 to ~1.7 MPa.

#### 4.1.9. Magnesium Ammonium Phosphate

Calcium phosphates bound with a chemical binder usually produce an acidic environment, whilst magnesium ammonium phosphate (struvite) (Mg_3_(PO_4_)_2_) scaffolds that were printed with an ammonium phosphate binder produce a neutral pH reaction during manufacture [93]. After a post printing immersion treatment, samples had a compressive strength between 2–7 MPa. MG63 cell viability on the struvite scaffold continued to increase up until day 10, however at levels significantly below that of the positive controls (60% of the cell culture dish viability). The lower cytocompatibility of the struvite scaffolds may be due to the partial dissolution of the struvite powder into the cell culture medium, which caused significantly higher Mg^2+^ and PO_4_^3−^ ion concentration, as well as significantly lower Ca^2+^ ion concentration. In a physiological environment, the surrounding fluid would be replenished more often, which would control surrounding ion concentrations and hence these effects might not be seen in vivo.

### 4.2. Synthetic Polymers

#### 4.2.1. Poly (dl-lactide-co-glycolide) (PLGA)

PLGA scaffolds were produced using a binder of ethanol, acetone, and de-ionized water that had a pore size of 1 mm, a porosity of 50%, a compressive strength of 7.8 ± 3.1 MPa, and a Young’s modulus of 77.2 ± 10.8 MPa [109]. The scaffolds were compared in vitro to a commercial open pore poly-lactic acid scaffold (OPLA^®^, Becton-Dickinson (BD) Inc., Franklin Lakes, NJ, USA) and a Collagen scaffold (Becton-Dickinson (BD) Inc.). The mechanical properties of the printed scaffold were 40 time higher than the OPLA^®^ scaffold and 18,000 times higher than the BD collagen scaffold. Cell viability of human fetal osteoblasts at 24 h was 95%, and after 48 h 81% when compared to the controls. There was no significant difference between the ALP activity of the cells on any of the scaffolds. In this work, cells were seeded with fibroin glue, which remained for the length of the experiment. Therefore, the similar cellular responses could be due to cellular interactions with the fibroin glue and not with the scaffold surfaces.

PLGA scaffolds created using the same method were implanted into the periosteum and iliac crest of rabbits [109]. When implanted into the periosteum, the scaffolds were encapsulated in a dense fibrous tissue with little tissue penetration. Scaffolds that were implanted in the iliac crest were encapsulated in a bone-like matrix that showed a high degree of integration between the scaffold and the native bone. These results highlight the dependence of the implantation site on the tissue response.

Simon et al. compared scaffold architecture on bony ingrowth using four different scaffolds with different porous architectures [110]. Implanted into rabbit trephine defects, the scaffolds were compared to a commercial coralline scaffold (Interpore). After 16 weeks, there was no significant difference in bone volume (around 50%) for each group, however linear bone ingrowth was significantly higher for the Interpore scaffold. All of the scaffolds were penetrated by fibrous tissues.

#### 4.2.2. Poly(e-caprolactone) (PCL)

Scaffolds composed of either pure PCL or a composite with a ratio of 50:50 PCL and β-TCP, were designed with 1 or 2 mm sized channels [111]. Seeded with porcine bone marrow-derived progenitor cells (pBMPC), there was no deformation or shrinkage for any of the scaffolds after two weeks in cell culture. At every time point, the PCL/β-TCP scaffold with 1 mm channels had significantly higher cell concentrations than the other scaffolds and it had the highest level of collagen formation.

### 4.3. Clinical Study

A long term clinical study has been completed using α-TCP printed facial bones and involved 20 patients [81,82,83]. The mechanical properties of the low temperature fabricated artificial bones was less than that of other sintered bone substitutes, however it was deemed sufficient for non-load bearing facial bones. After one year, 18 of 21 implant sites had formed a bony union between the implant and the native bone. The artificial bone did not change shape, although there was an average increase in thickness of 3.3%. Two adverse events were recorded during the first year, one caused by physical trauma, the other due to infection that had developed as a result of the patient being a carrier of MRSA. After the first year, three further adverse events occurred, in one case, more than five years after implantation. In cases where the implant failed, a gap was usually present between the artificial bone and the native bone immediately after surgery. By the time of the last follow up, all patients were satisfied with the aesthetics of the device after implantation. Overall, the clinical trial demonstrated the potential of powder bed inkjet printing for producing artificial bone implants.

## 5. Extrusion Printing

Extrusion printing can be split into two main techniques: the extrusion of molten material, referred to as fused deposition modelling (FDM) and the extrusion of gelling liquid material. The extrusion of molten materials requires the use of a thermoplastic that is usually fed to the printhead in the form of a long filament. Within the printhead, the filament is melted to a predetermined temperature and is extruded through the nozzle. As this method requires the use of a thermoplastic, its use has generally been limited to PCL and PLA for the research of biodegradable bone scaffolds. The accuracy and shape of the final structure is dependent on the speed at which the molten extruded filament cools down and hardens after it has been dispensed.

Another form of extrusion printing is based around the dispensing and gelling of liquid material. Extrusion is controlled via pressure based mechanisms, such as via a syringe or pneumatic pressure. This form of extrusion printing can use high-viscosity hydrogels (30 to $6\times 10^{8}$ mPa·s) extruded through micro-sized nozzles [112,113].

**Figure 7:**
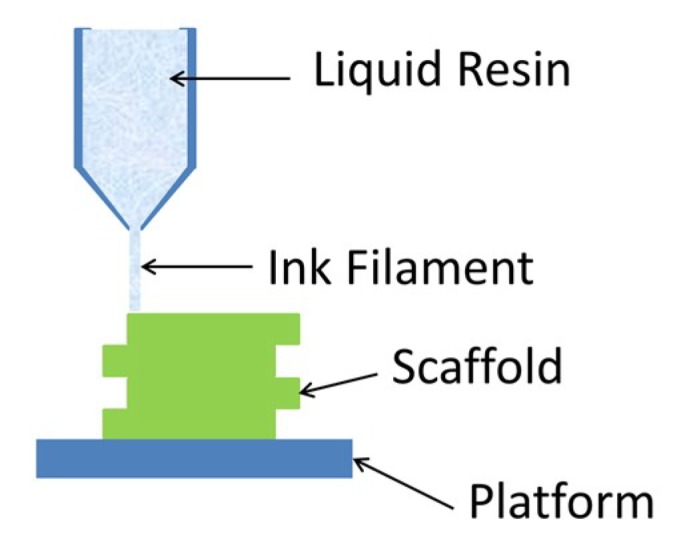
Schematic of extrusion based printing. A liquid resin is extruded in the form of a filament into a predetermined pattern.

Due to its fast processing speed and low startup costs, the application of this technology in fabricating scaffolds has greatly increased [114]. The disadvantages of extrusion printing include low resolution (around 200 μm [24]) and the requirement for high viscosity inks. The extrusion printing system can be pneumatic-based or mechanical based system, as demonstrated in Figure 7. A large variety of biomaterials can be used for this technology (compared in Table 4), including natural and synthetic polymers; ceramics can be printed as a paste, however are often used as low concentration additions to more easily printable polymeric hydrogels.

### 5.1. Bioceramics

#### 5.1.1. Hydroxyapatite

A comparison between low temperature fabrication (without sintering) and high temperature fabrication (with sintering) was performed for hydroxyapatite (HA) and Polyetheretherketone composite scaffolds [127]. Extrusion was performed using an ink of a HA slurry mixed with a PVA/PEG binder. It was found that low-temperature extrusion exhibited a uniform microstructure, whereas after sintering, micropores developed along the internal structure of the filaments that could be beneficial for cellular attachment.

#### 5.1.2. Tricalcium Phosphate (TCP)

Scaffolds for sinus augmentation procedures were fabricated using TCP and HA with a weight ratio of 70/30 [128]. Extrusion was performed with a ceramic slurry, and scaffolds were subsequently sintered at 1250 °C for 1 h. The scaffold had a mesh like structure with a filament diameter of 300 ± 25 μm and pore size of 370 ± 25 μm, resulting in a total porosity of about 60%. Complete tissue integration occurred after 45 days when implanted into adult sheep. The HA/TCP scaffold was well tolerated by the host tissue and it underwent a progressive process of tissue regeneration.

Scaffolds were fabricated by Boga et al. that had different ratios of tricalcium phosphate (TCP) and alginic acid (AA) (60:40, 70:30, 80:20) [124]. After printing, the AA was crosslinked by immersing the scaffolds overnight in a 5% CaCl_2_ solution. The presence of AA created a bone-like structure, increasing mechanical resistance and elasticity. With increased ceramic content, the scaffolds became brittle and displayed poor mechanical resistance. By incorporating the scaffolds with graphene oxide (GO), the Young’s modulus of scaffolds with a 60:40 TCP:AA ratio, increased from 154.4 ± 8.7 MPa to 188.3 ± 18.5 MPa. The addition of GO also controlled the swelling profile of the scaffold that had a large swelling ratio, which could otherwise lead to compressive pressures on the surrounding tissue and be a source of pain for the patient [124,129].

Martinez-Vazquez et al. used polymer infiltration to improve the mechanical strength of β-TCP scaffolds [125]. A β-TCP slurry was combined with Darvan^®^ C as a dispersant, and hydroxypropyl methylcellulose as a thickening agent. The scaffolds were subsequently sintered at 1200 °C for 1 h. Using a polymer melt, the scaffolds were infiltrated with either PCL or PLA, increasing the compressive strength of the β-TCP scaffold from 20 ± 2 MPa, to 60 ± 10 MPa, and 130 ± 20 MPa for a PCL and PLA coating, respectively.

Mandibular defects in rabbits were treated with β-TCP scaffolds with a 330 μm pore size [130]. For extrusion, a gel was prepared out of β-TCP powder, Darvan^®^ A as a dispersant, hydroxypropyl to increase viscosity, and polyethylenimine as a gelation agent. Scaffolds were sintered to burn the organic component out and to densify the structure. Printed samples showed excellent integration, with bony ingrowth occurring from both defect walls. A highly cellular and vascularized bone structure was observed in direct contact with the scaffold.

#### 5.1.3. Strontium (Sr) Composites

Strontium is an important factor for maintaining human tissue function, especially for bone down-regulating osteoclast and up-regulating osteoblast activity and it is involved in mineralization of new bone [131,132,133]. Deng et al. fabricated scaffolds from Sr_5_(PO_4_)_2_SiO_4_ (SPS) blended with sodium alginate, that after fabrication were sintered at 1450 °C to remove the alginate [134]. The scaffolds were implanted into osteochondral defects in rabbits. When compared to TCP scaffolds, SPS scaffolds produced higher proliferation rates and promoted greater subchondral bone regeneration.

To provide a highly porous structure whilst maintaining mechanical properties, Entezari et al. designed Sr-HT-Gahnite scaffolds with a hexagonal architecture [135]. With 50% porosity, the hexagonal architecture provided the scaffold with a compressive strength of 180 MPa, this was up to 42 MPa higher than scaffolds fabricated with conventional architectures. The structure was even able to produce a compressive strength of 90 MPa with 70% porosity.

### 5.2. Synthetic Polymers

#### 5.2.1. Polylactic Acid (PLA)

Gregor et al. compared the effect of porosity on mechanical properties of printed PLA scaffolds using a fused deposition modelling technique [116]. Scaffolds were fabricated with 30% and 50% porosity with respective pore sizes of 350 and 700 µm. The 30% porosity scaffold had a compressive Young’s modulus of 45.61 ± 11.80 MPa, which decreased to 29.96 ± 14.03 MPa with a 50% porosity. Although the higher porosity had a lower modulus, it promoted the greater proliferation and osteoconduction of osteosarcoma cells.

Grémare et al. also investigated the effect of pore size on scaffolds that were fabricated from PLA using FDM [136]. Three pore sizes were produced: 150, 200, and 250 µm. No significant differences in the ultimate tensile strength was observed between the three pore sizes, averaging around 8 N. Human bone marrow stromal cells (HBMSCs) that were seeded onto the scaffolds showed similar high cell viabilities.

The effect of increasing the strut spacing was investigated by Teixeira et al. [118]. Using an FDM printer, PLA scaffolds were printed with 0.8, 1.0, and 1.2 mm spacing between the struts, resulting in respective porosities of 55%, 60%, and 66%, and mechanical strengths of 13.25 ± 1.6 MPa, 9.47 ± 0.47 MPa, and 5.57 ± 0.27 MPa. Teixeira et al. also fabricated surface functionalized PLA scaffolds by coating them with collagen and polydopamine [117]. Seeded with porcine bone marrow stem cells, the functionalized scaffolds demonstrated better seeding efficiency and higher metabolic activity after seven days than that of the non-functionalized scaffolds.

Poly(l-lactic acid) (PLLA) combined with multiwalled carbon nanotubes (MWCNTs) enhanced scaffold mechanical properties [137]. PLLA/MWCNTs composite scaffolds with porosities between 65–70% were fabricated while using a pressure-assisted microsyringe. Elastic modulus increased with increasing MWCNTs concentration, up to 6.25 mg/mL, above which, the elastic modulus decreased rapidly. Scaffolds supported osteoblastic proliferation and activity in in vitro cell culture.

#### 5.2.2. Poly(dl-lactide-co-glycolide) (PLGA)

Kim et al. fabricated a PLGA/PCL scaffold using multi-head deposition system (MHDS) for a more rapid fabrication than with a single head printer [120]. The PLGA/PCL scaffold had a uniform pore size of 600 μm and the height of 200 μm. PLGA was blended with PCL to improve its brittle structure and poor mechanical properties, with final scaffold having 69.6% porosity and a compressive modulus of 12.9 MPa.

#### 5.2.3. Poly(e-caprolactone) (PCL)

Hutmacher et al., also performed mechanical testing on polycaprolactone (PCL) scaffolds fabricated with a porosity of 61% [121]. The compressive strength for the scaffold was 41.9 ± 3.5 MPa, however when soaked in phosphate buffer solution for one day, this dropped to 29.4 ± 4.0 MPa [121].

PCL scaffolds implanted in vivo have shown promising results. PCL scaffolds with a pore size of 500 μm and a porosity of 51.80% were implanted into critical sized bony defects in rabbit calvarium [138]. New bone formation was more prolific on the PCL scaffold as compared to a sham surgery control site. Only the porous area of the scaffold was able to regenerate with new bone over a period of 12 weeks, as the PCL degradation rate did not match the bone regeneration.

To treat an early stage of femoral head osteonecrosis, Kawai et al. have fabricated functionally graded scaffolds made of PCL and β-TCP [139]. The scaffolds were fabricated using FDM and a feeder “ink” filament consisting of both PCL and β-TCP. The scaffolds had a graduated porosity: 59.5 ± 1.2% in the middle, and 16.4 ± 1.7% in the proximal region, designed to closely mimic the anatomical porosity of a femoral head. The compressive strength of samples with 60% porosity was 2.2 MPa that increased to 9.5 MPa with 15% porosity. Implanted in rabbit femoral heads and assessed after eight weeks, the scaffolds exhibited excellent bone ingrowth.

Nyberg et al. studied the mechanical properties and in vitro response of PCL scaffolds with different mineral additives [140]. The scaffolds were fabricated with a pneumatic-based FDM printer, where the ink is melted in an ink reservoir and then extruded by pneumatic pressure. The scaffolds were made either from pure PCL, PCL/TCP, PCL/HA, PCL with natural HA (Bio-Oss^®^, Princeton, NJ, USA), or PCL with decellularized bone matrix (DBM). All composites had 70:30 ratio of PCL:mineral additives and a porosity of 60%. Compressive moduli recorded 51, 37, 83, and 32 MPa for pure PCL, PCL/TCP, PCL/HA, and PCL/DBM, respectively. Adipose-derived stem cells (ASCs) were seeded on to the scaffolds to study the in vitro osteogenic response of the composites. Calcium content increased significantly for all samples, except for pure PCL and PCL/HA. Collagen 1 and osteocalcin expressions increased 10-fold in samples with DCB and natural HA as compared to PCL samples, suggesting that natural organic/inorganic and inorganic composites may be advantageous in bone tissue engineering.

To enhance compressive strengths, Goncalves et al. combined hydroxyapatite (HA) and carbon nanotubes (CNT) in PCL [123]. Scaffolds were fabricated with interconnected pores with a size ranging between 450–700 µm and a total porosity of 57%. The inclusion of CNTs at 2 wt % increased the compressive strength from 2.0 to 5.5 MPa. However, the inclusion of CNTs into a PCL-HA matrix had limited effects on mechanical properties. A PCL-HA Scaffold (50:50 wt %) had a compressive strength between 40–50 MPa, whilst a PCL-HA-CNT scaffold had a compressive strength of 50 MPa. The inclusion of CNTs might have had a limited effect on mechanical properties for the PCL-HA scaffold, but it has been shown that the presence of CNTs can improve protein adsorption and consequently cell attachment [141,142,143].

Arafat et al. produced PCL/TCP scaffolds that were subsequently coated with either carbonated hydroxyapatite (CHA) or a CHA-gelatin composite [144], as it has been shown to be beneficial for scaffold/cell interaction [145]. Fabricated scaffolds had a honey comb structure with 100% interconnectivity and 65% porosity. Cultured with porcine bone marrow mesenchymal stem cells (BMSCs), the CHA-gelatin coated scaffolds had the fastest proliferation rates. The inclusion of CHA improved cell morphology and enhanced the stimulation of osteogenic differentiation of the BMSCs.

Extruded PCL/β-TCP scaffolds were used to reconstruct maxillofacial defects in dogs [146]. Scaffolds had a 500 × 100 μm pore size and 63% porosity. After seven months, the maxillary appearance was restored and appeared the same as the healthy side, demonstrating that extruded PCL/β-TCP scaffolds can maintain anatomical uniformity and appearance and provided optimal strength and function when applied in vivo.

Shanjani et al. demonstrated a sequential process involving two fabrication techniques to produce a hybrid scaffold made from a rigid porous PCL structure combined with a soft cured PEGDA hydrogel [147]. The rigid PCL provided the scaffold with mechanical strength, whilst the soft hydrogel can be used for incorporating cells and/or growth factors. The PCL was extruded using FDM whilst the hydrogel was crosslinked using SLA and an LAP photoinitiator. The hybrid scaffold had a compressive strength of 6 MPa. Human umbilical vein endothelial cells (HUVECs) incorporated into the scaffolds maintained high levels of viability.

#### 5.2.4. Poly(Propylene Fumarate) (PPF)

Trachtenberg et al. found that, by controlling the dispersion of nHA using sodium dodecyl sulfate (SDS) within a polypropylene fumarate (PPF) matrix, mechanical properties could be optimized [148]. The incorporation of SDS significantly decreased fiber diameter from 0.70 ± 0.1 mm to 0.63± 0.14 mm, and increased pore size from 0.45 ± 0.12 mm to 0.52 ± 0.13 mm. This resulted in porosities of 0.36 ± 0.01% without SDS and 0.49 ± 0.02% with SDS. However, compressive tests for both scaffolds exhibited similar moduli, around 49 MPa.

#### 5.2.5. Pluronic^®^ F-127 Hydrogel

Feilden et al., printed Pluronic^®^ F-127 hydrogel scaffolds with microscopic alumina platelets and submicron alumina powder [115]. The addition of alumina powder and platelets produced a network that produced a high Young’s modulus of 99.1 ± 0.6 GPa and a compressive strength of 50 MPa.

## 6. Transfer to Alveolar Bone Augmentation

Each of the reported AM techniques has demonstrated their own individual advantages and disadvantages for the development of scaffolds for bone tissue augmentation. Research involving SLA manufactured scaffolds has been dominated by the use of natural and synthetically based hydrogels. Although the naturally based hydrogels can offer excellent cellular interaction and biocompatibility, they suffer from poor compressive strengths. Compressive strengths can be significantly improved through the use of synthetically based hydrogels, however this comes at the expense of biocompatibility. Yet, the achieved compressive strengths are still too low for in-situ load bearing applications.

SLS was able to produce scaffolds with compressive strengths within the range of cortical bone [13]. However, SLS suffers from a poor resolution and has the potential for loose powder to become trapped within the printed pores of complex designs that could negatively affect scaffold design and tissue penetration.

Powder bed inkjet printed scaffolds had a range of mechanical properties that largely depended upon post printing processes. Most of the research has been conducted involving purely ceramic scaffolds that ultimately have brittle properties and low compressive strengths. By performing post-printing sintering, the scaffolds became denser, thereby improving the compressive strengths, however possibly at the expense of geometrical accuracy to the design of the scaffold. Although sintering helped improve the mechanical properties of the scaffolds, it also increased the content of slower degrading phases such as HA, which would ultimately increase the length of time that the scaffold remains.

Extrusion based printing has been used to print composites of polymer and ceramic that have produced scaffolds with the largest compressive strengths of all the AM techniques. These scaffolds had compressive strengths that are similar to that of cortical bone [13], and are capable of supporting a dental implant with a 0° abutment when under load [15]. Similar mechanical properties were also observed with SLS, however the ability of extrusion printing to incorporate natural polymers and bioactive molecules make it the most appealing for future development.

In translation to augmentation of the alveolar ridge, all of the AM techniques have shown potential. Although SLA and powder bed inkjet printed scaffolds have demonstrated mechanical properties not suited for load bearing applications, they could still be applied for augmentation of the alveolar ridge. Bone grafts that are placed in the alveolar ridge are not always required to provide immediate mechanical support. Dental implant placement can be performed as a two-step process: (1) ridge augmentation using a bone grafting material followed by a regeneration period of around 6–9 months; and, (2) and subsequent implant placement [2]. During the usual 6–9 months regeneration period, bone tissue should infiltrate the scaffold and provide increased mechanical stability to the alveolar ridge.

The choice of immediate implant placement or placement after a healing period is dependent upon patent specific requirements. If the quality and quantity of bone is sufficient to support the implant, then the implant can be inserted immediately, possibly with a slight adjustment of the bone augmentation. However, when the quality and quantity of bone is too poor for immediate implant placement, a larger bone augmentation procedure is required, and therefore requires a longer healing period before implant placement. Depending on the successful infiltration and development of bone tissue within the scaffold, SLA and inkjet-based scaffolds may be most suitable for two-stage ridge augmentation techniques due to the longer healing periods for tissue integration.

SLS and extrusion based scaffolds have so far produced the highest compressive strengths, which are similar to that of cortical bone [13]. Compressive strengths are also high enough to support an implant with a 0° abutment when under load [15]. Therefore, these scaffolds may be applicable for procedures involving simultaneous implant placement and ridge augmentation.

Polymer/bioceramic composites have shown advantageous results in AM bone tissue scaffolds. The polymer provides mechanical ductility, whilst the bioceramic provides osteoinductivity and enhances osteoconductivity. All four reported techniques have been successful in fabricating such composites, with great in vitro and in vivo outcomes [40,66,111,139]. Additive manufacturing offers repeatability between designs and the ability to match scaffold dimensions to that of the patient’s defect prior to surgery. Moreover, it has shown progressive and superior outcomes in vitro and in vivo, as compared to conventionally fabricated implants [49,135].

Biological studies have so far indicated the great potential for 3D printed scaffolds. Promising in vivo studies suggest that these highly tailorable scaffold designs could be successful for bone integration, yet more extensive studies are required for a full assessment of each printing technique and the materials used. As there have been relatively few clinical trials performed, it is difficult to predict the transition of 3D printed scaffolds from the lab to their incorporation into clinical practice. However, a clinical trial using α-TCP scaffolds demonstrated an excellent fit to the native bone, achieved through the combined use of patient CT data and AM, which was crucial for long the term success of the scaffold [81,82,83].

## 7. Conclusions

The use of additive manufacturing techniques in the development of substitute bone grafts has been demonstrated to be an attractive alternative to conventional techniques. Scaffolds can be tailored to suit patient requirements, such as by their mechanical properties and being designed porosity. As scaffolds are designed on a computer, patient CT data can be used to produce scaffolds that match the exact defect dimensions, thereby improving bony union between implant and host bone, as well as shortening surgical procedures, which would otherwise require that the bone block used for the augmentation be cut to size during surgery.

The greatest limitation of the research to date is the scarcity of preclinical in vivo and even clinical trials. It is therefore difficult to evaluate their full capabilities as well as their limitations. Nevertheless, the few clinical trials that are reported, as well as the in vivo studies, suggest that 3D printed scaffolds have great potential for the application of alveolar ridge augmentation.

## Figures and Tables

**Table 1:** Summary of SLA Scaffold Properties.

Material	Scaffold Mechanical Properties	Porosity (%)	Cell Viability	References
GelMA (Irgacure 2959)	30 kPa * at the highest degree of methacrylation	Non-porous	Highest cell viability was reported in the lowest concentration of GelMA, 80–95%	[29]
SilMA (LAP)	910 kPa ** at the highest percentage of Sil-MA	/	SilMA exhibited similar absorbance in a cck-8 assay as GelMA	[32]
GelMA:SF (Irgacure 2959)	Reached 75 kPa ** with the highest concentration of SF	41.8	Highest OD values was over 2.0, a little bit higher than the metabolic activity of pure GelMA	[30]
Chitosan:PEGDA (Irgacure 819)	~1000 kPa * at the highest concentration of PEGDA	Non-porous	Ratios of 1:5 and 1:10 of Chitosan:PEGDA exhibited the highest cell viability percentages 93–97%	[33]
Methacry. PCL/BG (Lucirin TPO-L)	3.4 MPa/dry * 2.5 MPa/wet *	63	Highest metabolic activity at highest BG concentration	[34]
CP CP/PCL	2.04 ± 0.12 MPa ** 4.55 ± 0.21 MPa **	/	CP:PCL had lower proliferation than pure CP but exhibited higher osteogenic markers expression	[35]
Methacrylated Alginate (VA-086)	3.3–12.4 kPa ** depending on the photoinitiator content	Non-porous	75% at moderate stiffness	[36]
Methacrylated PCL (Irgacure 2959, 369)	2.02 ± 2.87 MPa **	70.5 ± 0.8	Similar metabolic activity as in tissue culture polystyrene	[37]
PETMP:PEG-DVE	6.9 ± 1.8 MPa *	Non-porous	95% viability was exhibited in 120 h	[38]
Methacrylated PLA (camphorquinone)	9.43 ± 3.2 MPa with intermediate content of HA and highest content of TEGDMA (flexural strength)	/	Samples with highest TEGDMA and HA content exhibited better cell viability.	[39]
PTMC/HA (Lucirin TPO-L)	/	70	Addition of HA and TEGDMA promoted better cell attachment and proliferation	[40]

* Modulus, ** Strength. Abbreviations: β-TCP: β-tricalcium phosphate, BG: bioglass, GelMA: gelatin methacrylate, HA: hydroxyapatite, PEGDA: polyethylene glycol diacrylate, PEG-DVE: poly(ethylene glycol) divinyl ether, PETMP: pentaerythritol tetrakis(3-mercaptopropionate), PCL: polycaprolactone, PPF: polypropylene fumarate, SilMA: silk methacrylate, SF: silk fibroin, TEGDMA: triethylene glycol dimethacrylate.

**Table 2:** Summary of SLS Scaffold Properties.

Material	Scaffold Compressive Strength (MPa)	Porosity (%)	Biological Response	References
Magnesium silicate (Mg_2_SiO_4_)	40.29 ± 1.32 MPa	/	/	[61]
β-TCP/ZnO	17.89 MPa with 2.5 wt % ZnO	56.8	MG-63 cells indicated better attachment and proliferation with increased ZnO	[62]
HA/β-TCP	18.35 MPa with 30 wt % β-TCP	~61	MG-63 cells exhibited better attachment and morphology on scaffolds with 30 wt % and 50 wt %	[63]
PCL	2.3 MPa	50	Implanted in minipigs, exhibited full healing in 3 months	[64]
PCL/β-TCP	6 MPa * with 10 wt % β-TCP	68	Pure β-TCP exhibited better ingrowth than polymer/ceramic composite	[65]
PCL/HA	3.17 MPa with 15 wt % HA	70.31	PCL/HA scaffolds exhibited better bioactivity than pure PCL after 28 days	[66]
PHBV/CP	0.55 MPa with 15 wt % CP	62.6 ± 1.2	The incorporation of CP nanoparticles significantly improved cell proliferation and alkaline phosphatase activity	[67]
PLLA/CHA	Over 0.6 MPa with 10 wt % CHA	66.8 ± 2.5	Cellular response similar to pure PLLA	[67]

* Effective modulus. Abbreviations: β-TCP: β-tricalcium phosphate, HA: hydroxyapatite, PCL: polycaprolactone, PHBV: poly(hydroxybutyrate-co-hydroxyvalerate), CP: calcium phosphate, PLLA: poly(l-lactic acid), CHA: carbonated hydroxyapatite.

**Table 3:** Summary of Inkjet Printed Scaffold Properties.

Material	Binder	Porosity (%)	Compressive Strength (MPa)	Biological Response	References
HA	phosphoric acid 10% + 1 M NaH_2_PO_4_	60 59 *	1.9 ± 0.2 5.8 ± 0.3 *	Implanted into mice, there was minimal tissue penetration.	[84]
α-TCP	Sodium chondroitin sulfate 5% + disodium succinate 12%	61	18.6	Implanted into beagle dogs, experienced bony bridging, bone formation and the presence of bone marrow.	[85]
β-TCP	Phosphoric acid 1 wt %	/	2.5 7.5 *	Cranial plates implanted into mice, exhibited bone integration around edges and fibrous tissue in center.	[86]
β-TCP	Phosphoric acid 5 wt % Phosphoric acid 10 wt % Phosphoric acid 30 wt %	53 50 41	0.9 ± 0.1 3.0 ± 0.3 8.7 ± 1.3	When implanted, monetite and brushite degraded faster than the β-TCP	[87]
BCP (HA/β-TCP)					
100:0	PVA 0.6 wt %	42 ± 2	1.54 ± 0.13	Seeded with BMSCs, proliferation was highest for a HA/β-TCP ratio of 60:40 for both binder solutions. The scaffolds made with a PVA binder showed higher proliferation rates compared to the phosphoric acid samples.	
	Phosphoric acid 8.75 wt %	28 ± 2	2.81 ± 0.08		
20:80	PVA 0.6 wt %	44 ± 2	1.21 ± 0.11		
	Phosphoric acid 8.75 wt %	49 ± 3	2.36 ± 0.18		[88]
40:60	PVA 0.6 wt %	43 ± 3	1.26 ± 0.09		
	Phosphoric acid 8.75 wt %	47 ± 2	2.57 ± 0.23		
60:40	PVA 0.6 wt %	42 ± 1	1.35 ± 0.11		
	Phosphoric acid 8.75 wt %	49 ± 3	2.66 ± 0.20		
TTCP	Phytic acid 25 wt %	39.4 ± 1.5	1.3 ± 0.2 4.0 ± 0.4 *	/	[89]
Brushite	Phosphoric acid 20 wt %	38.8	23.4 ± 3.3	Showed good biocompatibility when seeded with osteoblastic cells.	[90]
	Phosphoric acid 20 wt %	45 29 *	5.3 ± 0.6 22.3 ± 1.5 *	When implanted, degraded slower than monetite samples, and over a 4-week period developed HA phases.	[87]
Monetite	Phosphoric acid 20 wt %	43.8	15.3 ± 1.1	Showed good biocompatibility when seeded with osteoblastic cells.	[90]
	Phosphoric acid 20 wt %	44	15 *	After 8 weeks implanted into rabbits, had completely integrated/resorbed into native bone.	[91]
Calcium Sulfate	2-pyrrolidinone	45.04	0.7 0.55 **	Samples were non-cytotoxic when sintered above 1000 °C.	[92]
Struvite	Ammonium phosphate	/	0.23 ± 1.37 7.01 ± 1.37 *	Osteoblastic cells showed good cell viability over 10 days.	[93]

* Immersion treatment, ** Sintered. Abbreviations: α-TCP—alpha-tricalcium phosphate, β-TCP: β-tricalcium phosphate, BCP: biphasic calcium phosphate, BMSCs: bone marrow mesenchymal stem cells, HA: hydroxyapatite, TTCP: tetracalcium phosphate.

**Table 4:** Summary of Extrusion Printed Scaffold Properties.

Material	Porosity (%)	Compressive Strength (MPa)	Biological Response	References
Pluronic F-127 hydrogel	/	50 MPa	/	[115]
PLA	40%	45.61 MPa	Metabolic activity and proliferation rate of osteosarcoma cells MG-63 did not have significant differences between each porosity.	[116]
PLA	50–60%	29.96 MPa		
PLA	60 ± 1.5%	9.47 MPa	Better seeding and metabolic activity with collagen/dopamine coating	[117]
PLA	55%	13.25 ± 1.6	Scaffolds with 66% porosity exhibited higher cell count	[118]
	60%	9.47 ± 0.47		
	66%	5.57 ± 0.27		
PBT	62.11 ± 0.36%	10.44 ± 2.09 MPa	/	[119]
PLGA/PCL	69.6%	12.9 MPa	Mesenchymal stem cells demonstrated good proliferation rates	[120]
PCL	61 ± 1%	Dry 41.9 ± 3.5 MPa Wet 29.4 ± 4.0 MPa	Human fibroblasts and osteoprogenitor cells proliferated, differentiated and deposited ECM	[121]
PCL	54.9%	/	Cardiomyoblasts attached to the structure, although a pore size of 250µm did not allow for cells to migrate.	[122]
PCL/HA/(0.2%) CNT PCL/HA	~40% 57%	/	Higher concentrations of CNT enhanced cell adhesion and spreading of MG-63 cells	[123]
TCP/AA 60/40-GO TCP/AA, 60/40	/ /	/	Over a 21-day period, human osteoblasts had secreted mineral deposits	[124]
β-TCP PCL/β-TCP PLA/β-TCP	49%	20 ± 2 MPa 60 ± 10 MPa 130 ± 20 MPa	/	[125]
Sr-HT-Gahnite	66.1%	53 ± 9 MPA	/	[126]
	52.1%	121 ± 12 MPa		
	48.5%	140 ± 15 MPa		

AA: alginic acid, CNT: carbon nanotube, PBT: polybutylene terephthalate, PCL: polycaprolactone, PLA: polylactic acid, PLGA: poly (DL-lactide-co-glycolide), PPF: poly (propylene fumarate).

## Abbreviations

2PP	Two-Photon Polymerization
AM	Additive Manufacture
AA	Alginic Acid
ALP	Alkaline Phosphatase
ASCs	Adipose-derived stem cells
BCP	Biphasic Calcium Phosphate
BG	Bioactive Glass S53P4
BMSCs	Bone Marrow Mesenchymal Stem Cells
CHA	Carbonated Hydroxyapatite
CP	Calcium Phosphate
CNT	Carbon Nanotubes
CPP	Calcium Polyphosphate
CS	Calcium Sulfate
DCP	Dicalcium Phosphate
EHA	2-ethylhexyl Acrylates
GBR	Guided Bone Regeneration
GelMA	Gelatin Methacrylate
GO	Graphene Oxide
HA	Hydroxyapatite
hFOB	Human Fetal Osteoblastic
HIPEs	High Internal Phase Emulsions
HUVEC	Human Umbilical Vein Endothelial Cells
IBOA	Isobornyl Acrylate
MWCNTs	Multiwalled Carbon Nanotubes
nHA	Nano-Hydroxyapatite
NVP	*N*-vinyl-2-pyrrolidone
OCP	Octacalcium Phosphate
pBMPC	Porcine Bone Marrow-Derived Progenitor Cells
PBT	Polybutylene terephthalate
PCL	Polycaprolactone
PDLLA	Poly(d,l-lactide)
PEGDA	Polyethylene Glycol Diacrylate
PEG-DVE	Poly(ethylene glycol) Divinyl Ether
PETMP	Pentaerythritol Tetrakis(3-mercaptopropionate)
PHB	Poly(hydroxybutyrate)
PHBV	Poly(hydroxybutyrate-co-hydroxyvalerate)
PLA	Polylactic Acid
PLGA	Poly(dl-lactide-co-glycolide)
PLLA	Poly(l-lactic acid)
PPF	Poly (propylene fumarate)
PTMC	Poly(trimethylene carbonate)
PVA	Poly Vinyl Alcohol
SDS	Sodium Dodecyl Sulfate
SF	Silk Fibroin
SilMA	Silk Methacrylate
SLA	Stereolithography
SLS	Selective Laser Sintering
Sr	Strontium
ß-TCP	Beta-Tricalcium Phosphate
TCP	Tricalcium Phosphate
TEGMA	Triethylene Glycol Dimethacrylate
TTCP	Tetracalcium Phosphate
